# Perinatal Outcomes of Newborns of COVID-19-Infected Pregnant Women: An Updated Systematic Review and Meta-Analysis

**DOI:** 10.7759/cureus.54306

**Published:** 2024-02-16

**Authors:** Khaled El-Atawi, Muzafar Gani Abdul Wahab, Yasser Elsayed, Maysa Saleh

**Affiliations:** 1 Pediatrics/Neonatal Intensive Care Unit, Latifa Women and Children Hospital, Dubai, ARE; 2 Pediatrics, McMaster University, Hamilton, CAN; 3 Pediatrics and Neonatology, Health Sciences Centre-Winnipeg, Max Rady College of Medicine, University of Manitoba, Manitoba, CAN; 4 Pediatrics and Child Health, Al Jalila Children's Hospital, Dubai, ARE

**Keywords:** newborn, covid-19, perinatal outcomes, vertical transmission, epidemic

## Abstract

In this systematic review and meta-analysis, we aimed to review the characteristics and outcomes of the newborns of Coronavirus disease 2019 (COVID-19) infected pregnant women. We conducted an online bibliographic search using the following electronic databases: MEDLINE via PubMed, Scopus, Web of Science, and Cochrane Central. Studies were deemed eligible if they recruited newborns from mothers with confirmed COVID-19 and reported the perinatal outcomes of neonatal COVID-19 cases. A total of 20 studies were included. Neonates born to mothers with positive COVID-19 results have been shown to have significantly lower birth weights (mean difference, MD = -48.54 g, p = 0.04), increased risks of fetal distress (odds ratio, OR = 1.76, p < 0.00001), respiratory distress (OR = 1.96, p = 0.006), premature birth (OR = 2.08, p < 0.00001), neonatal death (OR = 2.20, p = 0.004), and a lower 5-minute Apgar score (OR = 1.44, p = 0.02). Additionally, they were more likely to be admitted to the neonatal intensive care unit (NICU) (OR = 2.25, p = 0.007) and test positive for COVID-19 themselves (OR = 9.88, p = 0.03). However, other parameters, such as risks for malformations, mechanical ventilation, hypoglycemia, and sepsis, appeared to be comparable between the two groups. Maternal infection with COVID-19 during pregnancy is associated with several neonatal outcomes, some of which are adverse and others that do not show significant deviation from norms. While our meta-analysis clearly illustrates heightened risks associated with premature birth, reduced neonatal weight, and other challenges, it also emphasizes that not all neonatal outcomes can be directly attributed to maternal SARS-CoV-2 infection.

## Introduction and background

Since December 2019, SARS-CoV-2, the causative virus leading to COVID-19, has caused over 771.5 million infections and more than 6,972,152 deaths [1]. According to the criteria provided by Wu Z and McGoogan JM, the vast majority of pregnant women are asymptomatic or experience mild illness [2, 3]. Nonetheless, any infection during pregnancy carries the risk of complications. A recent analysis of obstetrical cases indicated that 3% of pregnant women infected with COVID-19 required intensive care [4]. Perinatal death, asphyxia, disseminated intravascular coagulation, rash, low birth weight, early and late-onset infections, pneumonia, respiratory distress, hypertension, gestational diabetes, preeclampsia, and premature rupture of membranes have been linked to COVID-19 infection during pregnancy [5-12]. Congenital viral infections can cause death, multisystem organ damage, sepsis, and permanent disability in newborns [5]. In ten infants delivered to women with COVID-19 infection, Zhu H et al. found evidence of impaired liver function, thrombocytopenia, respiratory distress, premature labor, and fetal distress; however, all the newborns tested negative for the virus. Vertical transmission of the virus is a rare occurrence, but many of the newborn problems are attributable to premature birth [6]. In a recent investigation, Zhang ZJ et al. found only four affected infants in China who presented between 30 hours and 17 days after delivery, indicating a nosocomial infection. These newborns had minor or no illnesses, did not require special care, and appeared to be in good health. Three of the infants were separated from their mothers shortly after delivery and were not breastfed [7]. The mechanisms of viral vertical transmission during pregnancy are not well understood [8, 9]. Possible routes of fetal infection include spread from the maternal bloodstream to fetal capillaries, ascending urogenital infections, infected maternal macrophages, and contact between maternal endothelium and cytotrophoblasts [10]. Finally, maternal infections can be passed on to newborns during delivery. In this systematic review and meta-analysis, we aimed to review the characteristics and outcomes of newborns born to mothers with a COVID-19 infection at the time of birth.

## Review

Materials and methods

Literature Search

This systematic review was performed according to the Preferred Reporting Items for Systematic Reviews and Meta-Analyses (PRISMA) [11].

Eligibility Criteria

We included studies that met the following inclusion criteria: (I) Population: Studies including pregnant women with clinically or laboratory-confirmed COVID-19; (II) Comparison: Studies including pregnant women with negative COVID-19 as a control group; (III) Outcomes: Studies assessing the prenatal or neonatal outcomes of infected vs. non-infected pregnant women; (IV) Study Design: Observational studies, including cohort, case-control, and cross-sectional studies. We excluded case reports and case series, reviews, book chapters, theses, editorials, and letters. Additionally, we excluded studies comparing pregnant women vs. non-pregnant women, pregnant women pre vs. during COVID-19 or lockdown, infected hospitalized women vs. non-hospitalized women, infected neonates vs. non-infected neonates, and mild COVID-19 cases vs. severe COVID-19 cases.

Information Source

A computerized search from 2019 to October 2023 was conducted on MEDLINE via PubMed, Web of Science, Cochrane Library, and Scopus using the following keywords: ("COVID-19" OR "2019-nCoV" OR "SARS-CoV-2") AND ("pregnant" OR "vertical transmission" OR "neonatal" OR "prenatal" OR "maternal").

Selection Process

After the database searches, all citations were imported into the EndNote X9 Windows version. Duplicate references resulting from database content overlap were identified and removed. Two independent reviewers (K.A and Y.E) screened the titles and abstracts of all unique citations against the predefined inclusion and exclusion criteria. Any disagreements between the two reviewers at this stage were resolved through discussion, or, if necessary, a third reviewer (M.S) was consulted. Studies that appeared to meet the inclusion criteria, or for which there was insufficient information in the title and abstract to make a clear decision, were advanced to full-text review. Again, two independent reviewers (M.E.H and M.H) assessed each full-text article to determine its eligibility. Disagreements at this stage were resolved through consultation with a third reviewer (M.S). The reference lists of all included studies were scanned to identify additional studies that might have been missed during the initial database searches. Any potentially relevant studies identified through this process were subjected to full-text review and included if they met the criteria.

Data Collection Process and Data Items

For studies that met the inclusion criteria, relevant data were extracted using a standardized data extraction form. This form was piloted on a subset of included studies and refined as needed. Data extracted included study characteristics (study ID, design, country, groups, sample size, inclusion criteria, conclusion), patient characteristics (age, race, body mass index, comorbidities, pregnancy-related conditions), and neonatal outcomes (weight, death, NICU admission, sepsis, fetal distress, respiratory distress, premature death, Apgar score, need for mechanical ventilation, continuous positive airway pressure (CPAP), or oxygen therapy).

Quality Assessment

The methodological quality of the included studies was evaluated using the Journal Impact Benchmark (JIB) tool, chosen for its comprehensiveness and applicability to case-control, cohort, and cross-sectional designs. Each study was meticulously appraised based on standardized criteria provided by the JIB tool, encompassing aspects such as study design, participant selection, exposure and outcome assessment, and statistical analysis.

Statistical Analysis

Data were pooled in pairwise comparisons when suitable. Continuous data were pooled as mean difference (MD) and 95% confidence interval, and dichotomous outcomes as odds ratio (OR) and 95% confidence interval. Statistical heterogeneity was assessed using the Chi-Square test, with its extent measured by the I-Square test. A random-effects model was used to estimate effect sizes. Sensitivity analysis was performed using a one-out model. Publication bias and the effect of small studies were assessed using funnel plot visualization. Review Manager (RevMan, Cochrane Collaboration) version 5.4 was used for pooling studies.

Results

Results of Database Searching

**Figure 1:**
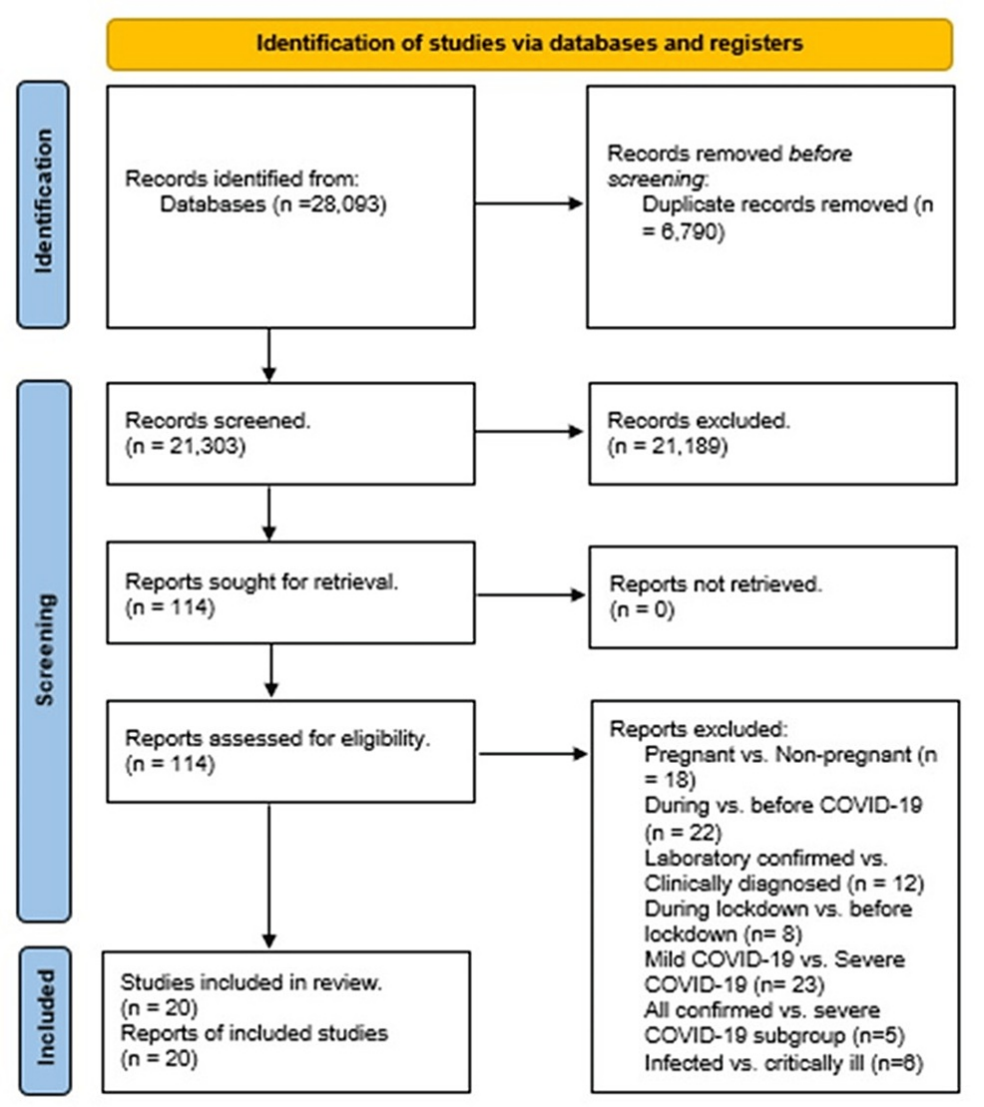
PRISMA flow diagram. PRISMA: Preferred reporting items for systematic review and meta-analysis.

The literature search yielded 14,247 articles from MEDLINE via PubMed, 5,062 articles from Scopus, 8,784 articles from Web of Science, and four articles from the Cochrane Library. Upon deduplicating these entries, 21,303 studies were retained for title and abstract assessment. This initial screening led to the exclusion of 21,189 studies. A subsequent in-depth review of the full texts was conducted for 114 articles, resulting in the final selection of 20 studies for qualitative synthesis and quantitative analysis [12-31]. The PRISMA flow diagram is presented in Figure 1.

Characteristics of Included Studies

A total of 20 studies were included from multiple countries, including the US, Sweden, China, Spain, India, Chile, France, Italy, Iran, and Romania. Four studies were case-control, one study was cross-sectional, and 15 studies were cohort studies. Notably, while many studies highlighted an increased risk of various pregnancy-related complications due to COVID-19, such as pre-eclampsia, preterm labor, fetal distress, premature rupture of membranes, venous thromboembolism, and other adverse maternal and neonatal outcomes [14, 15, 19, 20, 29, 30], several others found no significant association between the infection and severe complications [16, 22]. Specific findings also suggested that vaginal delivery could reduce the risk of intrapartum SARS-CoV-2 transmission and that the severity of maternal and neonatal outcomes might depend on the severity of the COVID-19 infection [26]. Table 1 summarizes the characteristics of the included studies.

**Table 1:** Characteristics of included studies. QA: Quality assessment; DIC: Disseminated intravascular coagulation; US: United States; QA: Quality assessment.

Study name	Country	Study design	Sample size	Criteria	Main findings	QA
Brandt et al., 2020 [12]	US	Case-control study	183	Pregnant women with confirmed COVID-19 infection by nasopharyngeal swab and PCR were admitted to the hospital from March 11 to June 11, 2020	COVID-19 infection during pregnancy increases the risk for adverse maternal and neonatal outcomes,	7
Dhuvetter et al., 2021 [13]	US	Cohort study	208	Any pregnant woman admitted to John H. Stroger Hospital tested for COVID-19 infection and included	Pregnant women at high risk for severe COVID-19 infection, only mild disease was observed.	9
Díaz-Corvillón et al., 2020 [14]	Chile	Cross-sectional study	583	Any pregnant women presenting in labor between April 27 and June 7, 2020, with no history of SARS CoV-2 disease during gestation	About half of the sample were asymptomatic cases. Universal screening is necessary in endemic areas.	8
Farghaly et al., 2020 [15]	US	Cohort study	79	Any pregnant woman admitted to Brookdale Hospital Medical Center tested for COVID-19 infection and included	Isolation precautions and vertical transmission prevention should done as most newborns are asymptomatic, and screening and follow-up of newborns for 14 days after birth.	11
Ferrazzi et al., 2020 [16]	Italy	Cohort study	42	Pregnant women who delivered during the study period with a confirmed diagnosis of COVID-19 infection prior to or within 36 hours after delivery	Vaginal delivery has a lower risk of intrapartum SARS-Cov-2 transmission to the newborn.	9
Gupta et al., 2021 [17]	India	Cohort study	3165	Any pregnant woman admitted to Shri Maharaja Gulab Singh maternity hospital for labor and delivery with no prior history of SARS‐CoV‐2 positivity.	COVID-19 infection increases the risk of maternal and perinatal adverse events.	9
Hcini et al., 2021 [18]	France	Cohort study	507	All pregnant women were admitted for delivery from June 16 to August 16, 2020, in the West French Guiana Hospital Center.	COVID-19 infection in pregnant women increases the risk of post-partum hemorrhage, transfusion, intra-uterine fetal demise, and admission to the ICU.	11
Katz et al., 2021 [19]	US	Cohort study	1454	Deliveries from 14 US medical centers, March 19-May 31, 2020	Symptomatic patients with COVID-19 infection have a higher risk for obstetric and neonatal complications	11
Cruz-Lemini et al., 2021 [20]	Spain	Cohort study	630	All asymptomatic obstetric patients detected by screening for SARSCoV-2 infection at admission to the delivery ward during the study period (March 23 to May 31, 2020)	Positive COVID-19 mothers have a higher risk for premature rupture of membranes than negative mothers.	9
Li et al., 2020 [21]	China	Case-control study	276	Any pregnant women admitted into the Hubei Provincial Maternal and Child Health Center, a tertiary hospital in Wuhan, from January 24 – February 29, 2020.	COVID-19 infection wasn't associated with any severe maternal and neonatal complications among pregnant women	7
Melguizo et al., 2021 [22]	Spain	Cohort study	2954	Any pregnant woman admitted to the hospital tested for COVID-19 infection and included between February 26 and November 5, 2020	COVID-19 infection in pregnant women increases the risk of preterm births, venous thromboembolism, and DIC.	10
Norman et al., 2021 [23]	Sweden	Cohort study	88159	All live-born infants delivered by women captured in the Swedish Pregnancy Register between March 11, 2020, and January 31, 2021	Maternal COVID-19 infection in pregnancy was significantly associated with small increases in some neonatal complications.	11
Steffen et al., 2021 [24]	US	Cohort study	1000	All delivering patients between May 1 and September 22, 2020, at the University of Iowa Hospitals and Clinics.	COVID-19 infection didn't increase the risk of any maternal or neonate complications	9
Tadas et al., 2021 [25]	India	Case-control study	362	All COVID-19-positive women who delivered in the Department of Obstetrics and Gynecology of Government Medical College, Nagpur, Maharashtra, from May 1, 2020, to August 31, 2020	The occurrence of maternal and neonatal adverse events depends on the severity of COVID-19 infection.	9
Taghavi et al., 2021 [26]	Iran	Case-control study	110	Pregnancies were referred to Shahid Mohammadi Hospital, Bandar Abbas, Hormozgan, Iran, between March and November 2020	COVID-19 increases the risk of preterm labor	9
Timircan et al., 2021 [27]	Romania	Cohort study	1039	Any pregnant woman given a single live child and tested for COVID-19 infection before or at admission	COVID-19 increases the risk of preterm labor, a premature rupture of membranes, and a lower APGAR score in newborns	11
Villar et al., 2021 [28]	Multicenter	Cohort study	2130	Any pregnant woman 18 years or older at any stage of pregnancy or delivery with the diagnosis of COVID-19 during the present pregnancy	COVID-19 infection in pregnant women increases the risk of severe maternal morbidity and mortality and neonatal complications	11
Abedzadeh-Kalahroudi et al., 2021 [29]	Iran	Cohort study	150	Any pregnant woman with confirmed COVID-19 infection and admitted to Shahid Beheshti Hospital. The control group was pregnant women who received prenatal care in midwifery clinics.	COVID-19 infection increases the risk of pregnancy-related conditions such as pre-eclampsia and preterm labor and the risk of fetal distress and prematurity of newborns	9
Adhikari et al., 2020 [30]	US	Cohort study	3374	Any woman gets tested for COVID-19 during pregnancy and delivered at Parkland Health and Hospital System.	COVID-19 infection was not associated with any increasing risk of pregnancy-related conditions.	9
Ahlberg et al., 2020 [31]	Sweden	Cohort study	759	Any pregnant woman coming into labor at Karolinska University Hospital, Stockholm, Sweden, from March 25 to July 24, 2020	SARS-CoV-2 test positivity in individuals in labor was associated with a higher prevalence of preeclampsia and a lower prevalence of induction of labor.	8

Quality Assessment

Based on the JIB tool, the quality of the four case-control studies and the fifteen cohort studies was deemed as "High." Similarly, the cross-sectional study by Díaz-Corvillón P et al. [14] showed high quality. The results of the quality assessment are presented in Table 1.

Characteristics of Included Patients

The total number of women with positive COVID-19 was 6,271, while the total number of those without COVID-19 was 109,850. The average age of the included patients was 29.88 years. Racial demographics, when reported, showed diverse populations, with notable representations from White non-Hispanic, Hispanic or Latina, and Black non-Hispanic groups, among others. BMI values, where specified, hovered around the mid-20s to low 30s, suggesting a mix of normal to overweight and obese populations. The prevalence of obesity varied across studies, with some indicating higher rates in the positive COVID-19 group than in the negative. Appendix 1 summarizes the demographics and pregnancy-related conditions of the included patients.

Neonatal Weight

**Figure 2:**
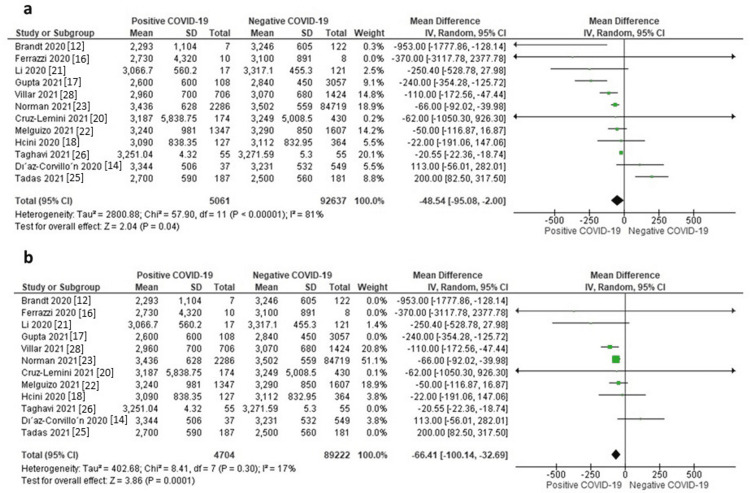
a) Forest plot of neonatal weight, b) Sensitivity analysis of neonatal weight.

**Figure 3:**
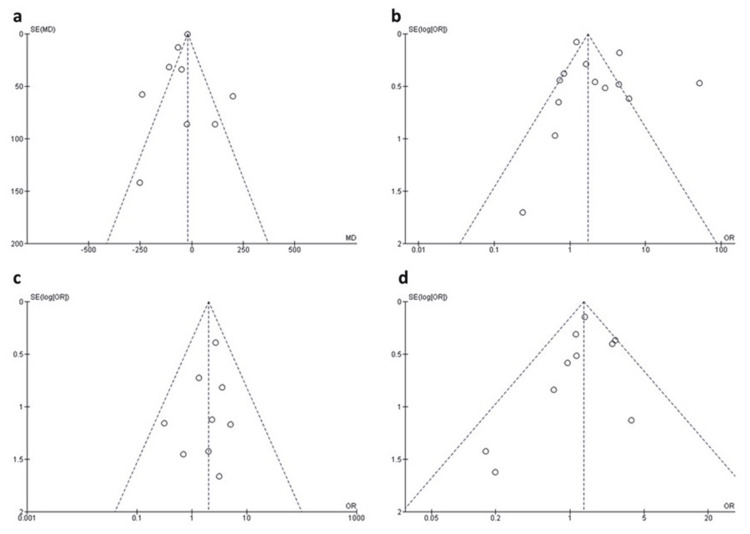
a) Funnel plot of neonatal weight, b) Funnel plot of NICU admission, c) Funnel plot of neonatal death, d) Funnel plot of Apgar 5 min (<7).

Twelve studies reported data on neonatal weight. The random-effects model showed that neonates of mothers with positive COVID-19 were associated with significantly lower weight compared to those of mothers with negative COVID-19 (MD = -48.54 g, 95% CI: -95.08 to -2.00, p = 0.04), as shown in Figure 2a. The pooled data were severely heterogeneous (I^2^ = 81%, p < 0.001). A sensitivity analysis was applied by excluding Gupta P et al., (2021) [17], Taghavi SA et al., (2021) [26], Tadas MP et al., (2021) [25], and Brandt JS et al., (2020) [12]. After excluding these studies from the analysis, the heterogeneity was resolved (I^2^ = 17%, p = 0.30), and the effect size remained significant (MD = -66.41 g, 95% CI: -100.14 to -32.69, p < 0.0001), as shown in Figure 2b. The funnel plot is presented in Figure 3a.

Fetal Distress

**Figure 4:**
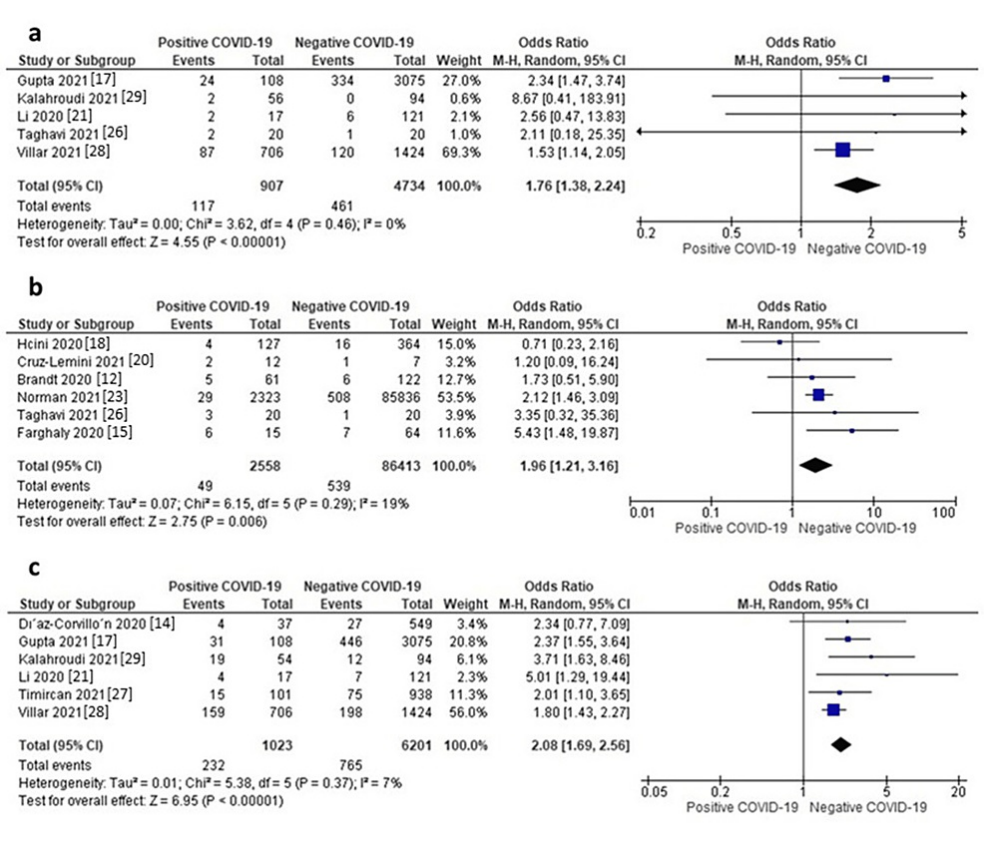
a) Forest plot of fetal distress, b) Respiratory distress, c) Premature death.

Five studies reported data on fetal distress. The random-effects model showed that neonates of mothers with positive COVID-19 were associated with a significantly higher risk of fetal distress compared to those of mothers without COVID-19 (OR = 1.76, 95% CI: 1.38 to 2.24, p < 0.00001), as shown in Figure 4a. Pooled data were homogeneous (I^2^ = 0%, p = 0.46).

Respiratory Distress

Six studies reported data on respiratory distress. The random-effects model showed that neonates of mothers with positive COVID-19 were associated with a significantly higher risk of respiratory distress compared to those of mothers without COVID-19 (OR = 1.96, 95% CI: 1.21 to 3.16, p = 0.006), as shown in Figure 4b. Pooled data were homogeneous (I^2^ = 19%, p = 0.29).

Premature Death

Six studies reported data on premature death. The random-effects model showed that neonates of mothers with positive COVID-19 were associated with a significantly higher risk of premature death compared to those of mothers without COVID-19 (OR = 2.08, 95% CI: 1.69 to 2.56, p < 0.00001), as shown in Figure 4c. Pooled data were homogeneous (I^2^ = 7%, p = 0.37).

NICU Admission

**Figure 5:**
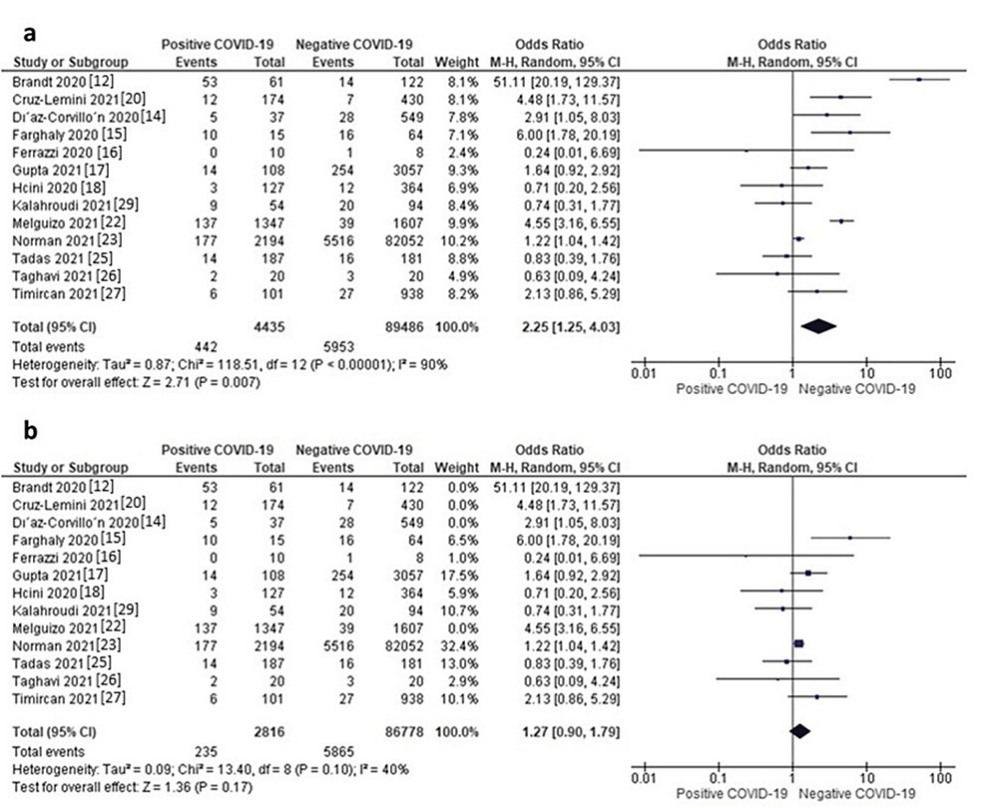
a) Forest plot of NICU admission, b) Sensitivity analysis of NICU admission.

Thirteen studies reported data on NICU admission. The random-effects model showed that neonates of mothers with positive COVID-19 were associated with a significantly higher risk of being admitted to NICU compared to those of mothers with negative COVID-19 (OR = 2.25, 95% CI: 1.25 to 4.03, p = 0.007), as shown in Figure 5a. The pooled data were highly heterogeneous (I^2^ = 90%, p < 0.00001). A sensitivity analysis was applied by excluding Brandt JS et al., 2020 [12], Cruz-Lemini M et al., 2021 [20], Díaz-Corvillón P et al., 2020 [14], and Cruz Melguizo S et al., 2021 [22]. After these studies were excluded from the analysis, the heterogeneity was reduced (I^2^ = 40%, p = 0.10); however, the effect size became insignificant (OR = 1.27, 95% CI: 0.90 to 1.79, p = 0.17), as shown in Figure 5b. The funnel plot is presented in Figure 3b.

Neonatal Death

**Figure 6:**
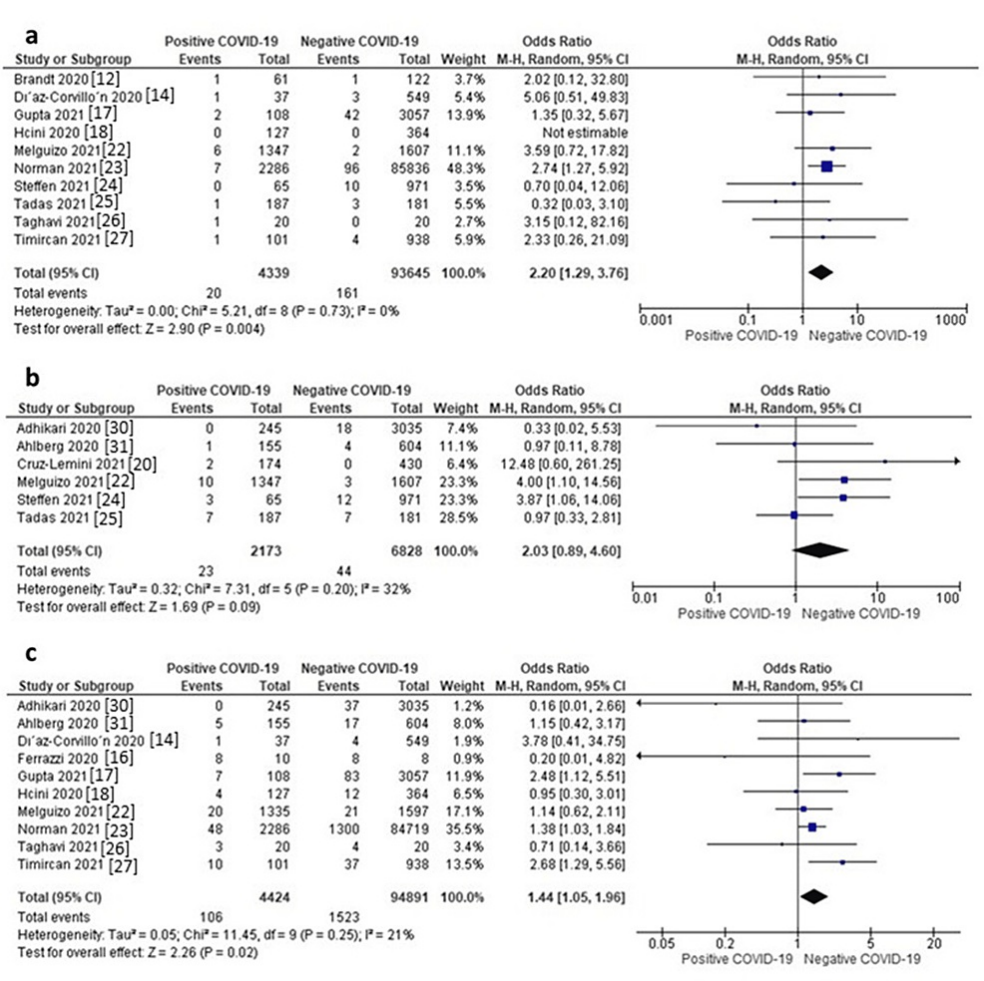
a) Forest plot of neonatal death, b) Forest plot of stillbirth, c) Forest plot of Apgar score <7.

Ten studies reported data on neonatal death. The random-effects model showed that neonates of mothers with positive COVID-19 were associated with a significantly higher risk of neonatal death compared to those of mothers without COVID-19 (OR = 2.20, 95% CI: 1.29 to 3.76, p = 0.004). Pooled data were homogeneous (I^2^ = 0%, p = 0.73), as shown in Figure 6a. The funnel plot is presented in Figure 3c.

Stillbirth

Six studies reported data on stillbirth. The random-effects model showed that neonates of mothers with positive COVID-19 were associated with a non-significantly higher risk of stillbirth compared to those of mothers without COVID-19 (OR = 2.03, 95% CI: 0.89 to 4.60, p = 0.09), as shown in Figure 6b. Pooled data were homogeneous (I^2^ = 32%, p = 0.20).

Apgar 5 min (<7)

Ten studies reported data on the 5-minute Apgar score (<7). The random-effects model showed that neonates of mothers with positive COVID-19 were associated with a significantly higher risk of having 5-minute Apgar scores less than 7 compared to those of mothers without COVID-19 (OR = 1.44, 95% CI: 1.05 to 1.96, p = 0.02), as shown in Figure 6c. Pooled data were homogeneous (I^2^ = 21%, p = 0.25). The funnel plot is presented in Figure 3d.

Gestational Age

**Figure 7:**
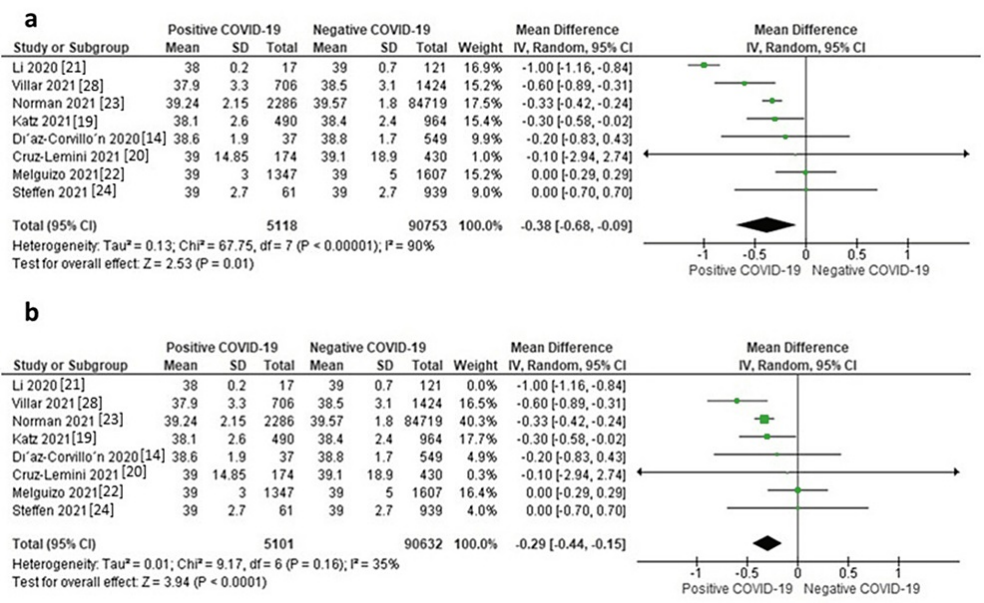
a) Forest plot of gestational age, b) Sensitivity analysis of gestational age.

Eight studies reported data on gestational age. The random-effects model showed that neonates of mothers with positive COVID-19 were associated with a significantly lower gestational age compared to those of mothers with negative COVID-19 (MD = -0.38 months, 95% CI: -0.68 to -0.09, p = 0.01). The pooled data were highly heterogeneous (I^2^ = 90%, p < 0.00001). A sensitivity analysis was applied by excluding Li et al., 2020. After this study was excluded from the analysis, the heterogeneity was resolved (I^2^ = 35%, p = 0.16), and the effect size remained significant (MD = -0.29 months, 95% CI: -0.44 to -0.15, p < 0.0001), as shown in Figure 7.

Small for Gestational Age

Seven studies reported data on small for gestational age. The random-effects model showed that neonates of mothers with positive COVID-19 were associated with a non-significantly higher risk of being small for gestational age compared to those of mothers without COVID-19 (OR = 1.14, 95% CI: 0.97 to 1.34, p = 0.11), as shown in Figure 7a. Pooled data were homogeneous (I^2^ = 0%, p = 0.98).

Low Birth Weight

Four studies reported data on low birth weight. The random-effects model showed that neonates of mothers with positive COVID-19 were associated with a significantly higher risk of low birth weight compared to those of mothers without COVID-19 (OR = 1.86, 95% CI: 1.27-2.72, p = 0.001), as shown in Figure 7b. Pooled data were homogeneous (I^2^ = 13%, p = 0.33).

Neonatal Positive COVID-19

Three studies reported data on neonatal-positive COVID-19. The random-effects model showed that neonates of mothers with positive COVID-19 were associated with a significantly higher risk of testing positive for COVID-19 compared to those of mothers without COVID-19 (OR = 9.88, 95% CI: 1.31 to 74.66, p = 0.03). Pooled data were moderately heterogeneous (I^2 = 63%, p = 0.07). After excluding the study by Norman M et al., 2021, the heterogeneity was resolved (I^2^ = 0%, p = 0.78), and the effect size remained significant (OR = 35.01, 95% CI: 4.24-288.84, p = 0.001).

Malformations

**Figure 8:**
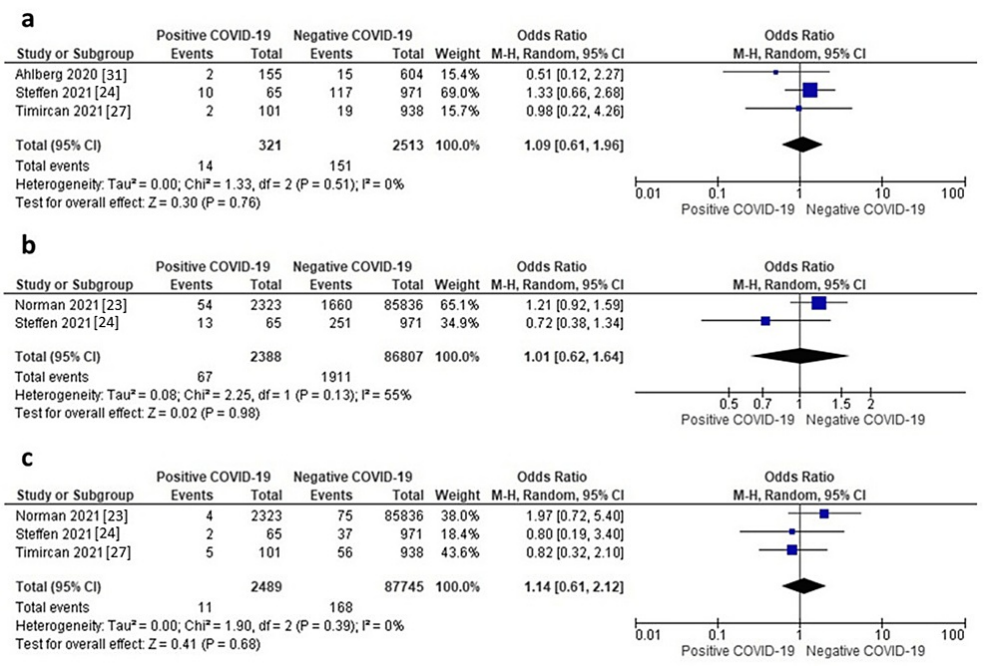
a) Forest plot of malformation, b) Forest plot of hypoglycemia, c) Forest plot of sepsis.

Three studies reported data on malformations. The random-effects model showed that neonates of mothers with positive COVID-19 had a comparable risk of malformations compared to those of mothers without COVID-19 (OR = 1.09, 95% CI: 0.61-1.96, p = 0.76), as shown in Figure 8a. Pooled data were homogeneous (I^2^ = 0%, p = 0.51).

Hypoglycemia

Two studies reported data on hypoglycemia. The random-effects model showed that neonates of mothers with positive COVID-19 had a comparable risk of hypoglycemia compared to those of mothers without COVID-19 (OR = 1.01, 95% CI: 0.62-1.64, p = 0.98), as shown in Figure 8b. Pooled data were homogeneous (I^2^ = 55%, p = 0.13).

Sepsis

Three studies reported data on sepsis. The random-effects model showed that neonates of mothers with positive COVID-19 had a comparable risk of sepsis compared to those of mothers without COVID-19 (OR = 1.14, 95% CI: 0.61-2.12, p = 0.68), as shown in Figure 8c. Pooled data were homogeneous (I^2^ = 0%, p = 0.39).

Mechanical Ventilation

**Figure 9:**
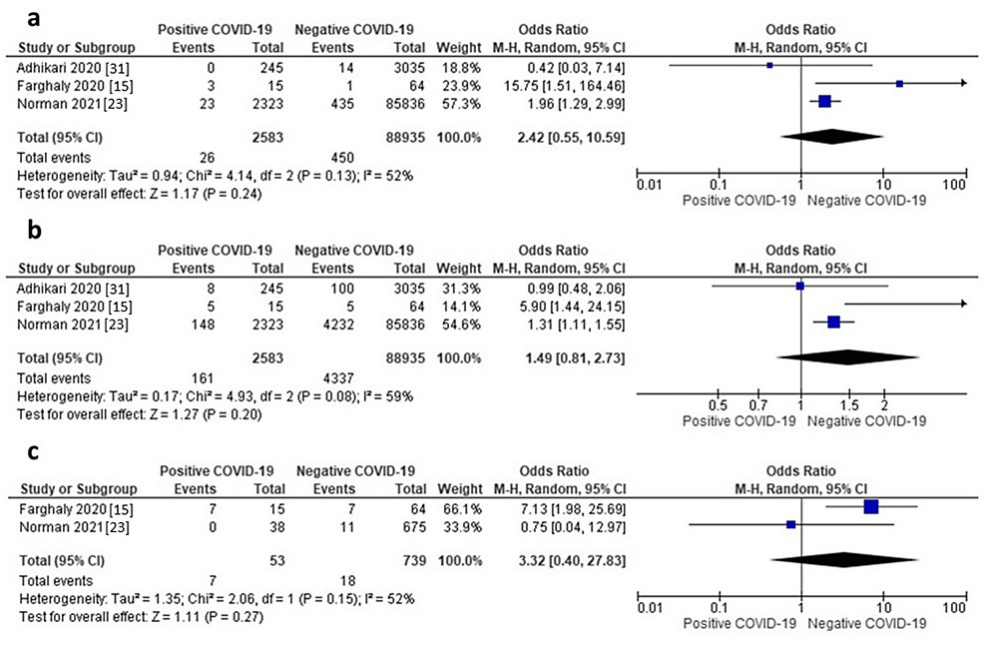
a) Forest plot of mechanical ventilation, b) Forest plot of CPAP, c) Forest plot of oxygen therapy. CPAP: Continuous positive airway pressure.

Three studies reported data on mechanical ventilation. The random-effects model showed that neonates of mothers with positive COVID-19 were associated with an insignificantly higher risk of mechanical ventilation compared to those of mothers without COVID-19 (OR = 2.42, 95% CI: 0.5-0.59, p = 0.24), as shown in Figure 9a. Pooled data were homogeneous (I^2^ = 52%, p = 0.13).

CPAP

Three studies reported data about CPAP. The random-effects model showed that neonates of mothers with positive COVID-19 were associated with an insignificantly higher risk of CPAP compared to those of mothers without COVID-19 (OR = 1.49, 95% CI: 0.81-2.73, p = 0.20), as shown in Figure 9b. Pooled data were moderately heterogeneous (I^2^ = 59%, p = 0.08).

Oxygen Therapy

Two studies reported data on oxygen therapy. The random-effects model showed that neonates of mothers with positive COVID-19 were associated with an insignificantly higher risk of oxygen therapy compared to those of mothers without COVID-19 (OR = 3.32, 95% CI: 0.40-27.83, p = 0.27), as shown in Figure 9c. Pooled data were homogeneous (I^2^ = 52%, p = 0.15).

Discussion

In this systematic review and meta-analysis, we assessed the potential effects of maternal COVID-19 positivity on various neonatal outcomes. The findings indicate that neonates born to mothers with positive COVID-19 results have significantly lower neonatal weight, higher risks of fetal distress, respiratory distress, premature death, reduced gestational age, and an increased likelihood of a 5-minute Apgar score below 7. Moreover, they face significantly higher risks of being diagnosed with low birth weight and testing positive for COVID-19. However, certain outcomes, such as malformations, hypoglycemia, sepsis, mechanical ventilation, and CPAP, showed no significant difference between groups, suggesting that while COVID-19 in expectant mothers may present specific neonatal challenges, not all observed outcomes can be attributed directly to maternal infection.

The impact of the current COVID-19 epidemic on pregnant women and their newborns is a critical aspect. Numerous studies have now been published on the negative effects of COVID-19 on pregnant women, their infection status, and the clinical features of their newborn babies. Their findings revealed that pregnant women with confirmed COVID-19 had a greater rate of preterm birth; however, this was not necessarily due to severe maternal respiratory illness. There was no indication of intrauterine transmission of COVID-19 when the infants' outcomes from the group of pregnant women with COVID-19 were examined [3, 32-34].

To investigate the possibility of vertical transmission, Moreno and his colleagues conducted a retrospective study of pregnant women diagnosed with COVID-19 (positive COVID-19 rRT-PCR) during the third trimester. Their findings demonstrated no detected cases of COVID-19 in the delivered neonates; however, 62% of them were admitted to a NICU, 46.1% were premature, and the mean period of NICU stay was 5.5 ± 6.4 days [35]. Many studies have also suggested no vertical transmission of COVID-19 [36-39], including a systematic review and meta-analysis of six studies [40]. A neonate delivered to a pregnant woman with COVID-19 tested positive for COVID-19 in a pharyngeal swab sample 36 hours after birth. Still, the qRT-PCR screening was negative for COVID-19 when taken from the placenta and cord blood, implying that intrauterine vertical transmission did not occur [41, 42]. Two articles highlighted the probability of vertical transmission of COVID-19 due to the presence of IgM antibodies in blood obtained from three neonates delivered to women with COVID-19, while the respiratory samples were all negative for COVID-19 [43, 44].

Our meta-analysis showed that newborns from COVID-19-positive mothers had a significantly higher incidence of premature death, which is the most prevalent unfavorable pregnancy outcome in COVID-19 patients. A meta-analysis conducted by Di Mascio et al. showed that the incidence of preterm birth was 41.1% in pregnant patients with COVID-19; of these, 25% were less than 37 weeks, and 19.6% were less than 34 weeks [40]. In a recent cohort, preterm birth before 37 weeks was found in 38.1% [35]. Future studies should include a larger sample of patients from other centers to detect variations in preterm delivery rates and determine whether premature birth is a significant adverse outcome in women with COVID-19 infection.

Similar to our findings, a previous meta-analysis of individual participants' data showed that neonates delivered by mothers infected with COVID-19 were found to have a heightened likelihood of NICU admission post-birth (RR = 1.86, 95% CI: 1.12-3.08). There was also an increased risk of these infants being born prematurely (RR = 1.71, 95% CI: 1.28-2.29) and a particularly pronounced risk for moderate preterm birth (RR = 2.92, 95% CI: 1.88-4.54). Furthermore, they showed an elevated risk of low birth weight (RR = 1.19, 95% CI: 1.02-1.40) [45].

In terms of stillbirth, we could not find a significant difference between both groups. Similarly, a previous meta-analysis showed no correlation between maternal infection and stillbirth [45]. Contrary to our findings, a systematic review observed that women with COVID-19 were 2.84 times more likely to experience stillbirth than those uninfected, based on a small sample of 35 stillbirths, with nine from the COVID-19 cohort [46]. Furthermore, a comprehensive study involving over 340,000 pregnancies in England reported an elevated risk of stillbirth with an adjusted OR of 2.17 (95% CI: 1.96-2.42) [47]. Potential discrepancies in results might arise from various analytical approaches. For instance, our definition of stillbirth was any fetal demise from 28 weeks' gestational age onwards [48], whereas some studies set an earlier gestational age. Given the rarity of stillbirth, further insights are essential to gauge potential risks and discern if risk fluctuations occur based on COVID-19 infection timing and severity.

Interestingly, our study did not identify any association between COVID-19 infection during pregnancy and neonates being categorized as small for their gestational age. This observation aligns with the results from the INTERCOVID study and a previous meta-analysis, which reported comparable outcomes [20, 45]. Thus, the combined results hint at a lack of correlation between COVID-19 infection during pregnancy and intrauterine growth restriction. However, it would be prudent to delve deeper into this issue, especially considering the potential impact of the timing and intensity of the infection during gestation.

Clinical implications

The findings of our review have pivotal clinical implications. Health professionals should be vigilant when providing prenatal and postnatal care to mothers infected with COVID-19, ensuring both maternal and neonatal health are paramount. It is essential to have a thorough risk assessment, which can guide timely interventions, thereby potentially reducing adverse outcomes. Clinicians should also be updated about the potential risks and effects of COVID-19 during pregnancy to offer the best possible care.

Future directions

The effects of maternal COVID-19 infection on neonates warrant further exploration, especially in larger cohorts and across different regions. Future research should focus on longitudinal studies that track neonatal outcomes over the long term, understanding the developmental and health implications as they grow. Moreover, the specific mechanisms underlying the observed outcomes remain elusive and merit further investigation. Studies examining the effects of maternal vaccination on neonatal outcomes will also be invaluable as we progress in our fight against the pandemic.

Limitations

While our meta-analysis provides critical insights, it is not devoid of limitations. The number of studies included was relatively small, which limits the comprehensiveness of our findings. We observed considerable heterogeneity in some pooled analyses, which can affect the generalizability of our conclusions. The inability to perform a subgroup analysis due to the lack of available studies further restricts our understanding. Additionally, variations in study design, population demographics, and clinical settings among the included studies might have introduced biases, potentially impacting the robustness of our results. Further studies are required to examine the association between the timing of infection (first, second, and third trimester) and the complication rate. In addition, the causes of neonatal death or mortality were not well investigated in the included studies.

## Conclusions

Maternal infection with COVID-19 during pregnancy presents with several neonatal outcomes, some of which are adverse and others that do not show significant deviation from the norms. While our meta-analysis clearly illustrates heightened risks associated with premature birth, reduced neonatal weight, and other challenges, it also emphasizes that not all neonatal outcomes can be directly attributed to maternal SARS-CoV-2 infection. Discrepancies observed in outcomes across various studies underscore the intricate nature of the virus's impact on neonatal health and the importance of considering factors like timing, severity, and the presence of other confounding variables.

## Appendices

Appendix 1 

**Table 2:** Demographics and pregnancy-related conditions of the included patients. DM: Diabetes mellitus; HTN, Hypertension; CVD: Cardiovascular disease.

Study Name	Groups	Comorbidity, [n(%)]						Pregnancy related conditions						Abortuses, [n (%)]	Nulliparous, [n (%)]	Multiple pregnancies, [n (%)]	Primipara, [n (%)]	Multipara, [n (%)]	Parity, [Mean (SD)]	Gravida, [Mean (SD)]	Type of delivery, [n (%)]		Neonate sex [n (%)]		Gestational age at childbirth (weeks)		Birth weight, g [Mean (SD)]	Birth height, cm [Mean (SD)]	Gestational age at delivery, week [ Mean (SD)]	Gestational age at dignosis, week [ Mean (SD)]	Smoking	
		DM	HTN	CVD	Renal disease	Asthma	Anemia	Gestational DM, [n (%)]	Pregnancy induced hypertension	Anemia	Premature labor	Cholestasis of pregnancy	Small for gestational age								Vaginal	CS	Male	Female	<37	>37					Smoker	Non-smoker
Abedzadeh-Kalahroudi et al., 2021 [29]	Positive COVID-19	9 (16.1)	6 (10.7)	NR	NR	NR	NR	NR	NR	NR	NR	NR	NR	NR	NR	NR	19 (33.9)	37 (66.1)	NR	2.39 (1.1)	18 (32.7)	37 (67.3)	27 (49.1)	28 (50.9)	19 (34.5)	36 (65.5)	3084 (512)	49.2 (2.3)	38.2 (1.35)	NR	NR	NR
	Negative COVID-19	21 (22.3)	11 (11.7)	NR	NR	NR	NR	NR	NR	NR	NR	NR	NR	NR	NR	NR	21 (22.3)	73 (77.7)	NR	2.48 (1.5)	49 (52.1)	45 (47.9)	46 (48.9)	48 (51.1)	12 (12.8)	82 (87.2)	2942 (679)	49.2 (4.2)	37.1 (3.1)	NR	NR	NR
Adhikari et al., 2020 [30]	Positive COVID-19	1 (0.4)	12 (5)	NR	NR	NR	NR	14 (6)	NR	NR	NR	NR	NR	7 (3)	73 (29)	6 (2)	NR	NR	NR	NR	NR	NR	NR	NR	NR	NR	NR	NR	NR	NR	NR	NR
	Negative COVID-19	57 (1.8)	145 (5)	NR	NR	NR	NR	207 (7)	NR	NR	NR	NR	NR	87 (3)	962 (31)	48 (2)	NR	NR	NR	NR	NR	NR	NR	NR	NR	NR	NR	NR	NR	NR	NR	NR
Ahlberg et al., 2020 [31]	Positive COVID-19	NR	NR	NR	NR	NR	NR	NR	NR	NR	NR	NR	NR	NR	60 (38.7)	3 (1.9)	NR	NR	NR	NR	NR	NR	NR	NR	NR	NR	NR	NR	NR	NR	4 (2.6)	147 (94.8)
	Negative COVID-19	NR	NR	NR	NR	NR	NR	NR	NR	NR	NR	NR	NR	NR	247 (40.9)	7 (1.2)	NR	NR	NR	NR	NR	NR	NR	NR	NR	NR	NR	NR	NR	NR	22 (3.6)	566 (93.7)
Brandt et al., 2020 [12]	Positive COVID-19	7 (11.5)	2 (3.3)	NR	1 (1.6)	2 (3.3)	2 (3.3)	NR	NR	NR	NR	NR	NR	NR	NR	NR	NR	NR	2 (1-3)	3 (2-4)	NR	NR	NR	NR	NR	NR	NR	NR	NR	NR	NR	NR
	Negative COVID-19	20 (16.4)	6 (4.9)	NR	0	4 (3.3)	4 (3.3)	NR	NR	NR	NR	NR	NR	NR	NR	NR	NR	NR	1 (1-3)	2 (2-4)	NR	NR	NR	NR	NR	NR	NR	NR	NR	NR	NR	NR
Dhuvetter et al., 2021 [13]	Positive COVID-19	2 (8.7)	4 (17.4)	NR	NR	4 (17.4)	NR	1 (4.3)	2 (8.7)	NR	NR	2 (8.7)	1 (4.3)	NR	NR	NR	NR	NR	1.7 (0-7)	3.1 (1-8)	NR	NR	NR	NR	NR	NR	NR	NR	37.9 (29-40)	33.2 (17-40)	NR	NR
	Negative COVID-19	8 (4.3)	25 (13.5)	NR	NR	24 (13.0)	NR	11 (5.9)	45 (24.3)	NR	NR	4 (2.2)	6 (3.2)	NR	NR	NR	NR	NR	0.8 (0-9)	2.4 (1-14)	NR	NR	NR	NR	NR	NR	NR	NR	39.8 (15-41)	37.1 (15-41)	NR	NR
Díaz-Corvillón et al., 2020 [14]	Positive COVID-19	1 (2.7)	0	NR	NR	0	NR	NR	NR	NR	NR	NR	NR	NR	NR	0	19 (51.3)	NR	NR	NR	NR	NR	NR	NR	NR	NR	NR	NR	NR	NR	0	NR
	Negative COVID-19	9 (1.7)	8 (1.5)	NR	NR	3 (0.6)	NR	NR	NR	NR	NR	NR	NR	NR	NR	3 (0.6)	266 (48.7)	NR	NR	NR	NR	NR	NR	NR	NR	NR	NR	NR	NR	NR	2 (0.4)	NR
Farghaly et al., 2020 [15]	Positive COVID-19	NR	NR	NR	NR	NR	NR	NR	NR	NR	NR	NR	NR	NR	NR	NR	NR	NR	NR	NR	5 (33)	10 (67)	3 (25)	12 (75)	2 (13.3)	13 (86.7)	2800 (1000)	NR	37.40 (3.8)	NR	0	NR
	Negative COVID-19	NR	NR	NR	NR	NR	NR	NR	NR	NR	NR	NR	NR	NR	NR	NR	NR	NR	NR	NR	37 (58)	27 (42)	28 (43.75)	36 (56.25)	3 (4.6)	61 (95.4)	3100 (500)	NR	38.67 (1.8)	NR	2 (3.1)	NR
Ferrazzi et al., 2020 [16]	Positive COVID-19	NR	NR	NR	NR	NR	NR	2 (20)	NR	NR	NR	NR	NR	NR	4 (40)	NR	NR	NR	NR	NR	NR	NR	NR	NR	5 (50)	5 (50)	2730 (840-4040)	NR	NR	NR	NR	NR
	Negative COVID-19	NR	NR	NR	NR	NR	NR	NR	NR	NR	NR	NR	NR	NR	2 (25)	NR	NR	NR	NR	NR	NR	NR	NR	NR	1 (12)	7 (88)	3100 (2770-3430)	NR	NR	NR	NR	NR
	Vaginal delivery	NR	NR	NR	NR	NR	NR	4 (17)	NR	NR	NR	NR	NR	NR	9 (38)	NR	NR	NR	NR	NR	NR	NR	NR	NR	5 (22)	18 (78)	3226 (2450-3740)	NR	NR	NR	NR	NR
Gupta et al., 2021 [17]	Positive COVID-19	NR	NR	NR	NR	NR	NR	NR	NR	NR	NR	NR	NR	NR	45 (41.6)	NR	NR	63 (58.3)	NR	NR	45 (41.6)	63 (58.3)	NR	NR	NR	NR	2600 (600)	NR	36.6 (3.3)	NR	NR	NR
	Negative COVID-19	NR	NR	NR	NR	NR	NR	NR	NR	NR	NR	NR	NR	NR	1542 (50.44)	NR	NR	1515 (49.56)	NR	NR	2143 (70.1)	914 (29.8)	NR	NR	NR	NR	2840 (450)	NR	37.5 (2.2)	NR	NR	NR
Hcini et al., 2021 [18]	Positive COVID-19	0	2 (1.4)	1 (0.72)	NR	0	5 (3.6)	13 (9.4)	15 (10.9)	11 (8.0)	7 (5.1)	NR	3 (2.1)	NR	30 (21.9)	0	NR	NR	2 (1-5)	3 (2-6)	NR	NR	NR	NR	NR	NR	NR	NR	NR	NR	NR	NR
	Negative COVID-19	5 (1.35)	6 (1.6)	2 (0.5)	NR	7 (1.8)	7 (1.8)	30 (8.1)	31 (8.3)	39 (10.5)	16 (4.3)	NR	6 (1.6)	NR	94 (25.4)	5 (1.4)	NR	NR	2 (0-4)	3 (2-6)	NR	NR	NR	NR	NR	NR	NR	NR	NR	NR	NR	NR
Katz et al., 2021 [19]	Positive COVID-19	10 (2.0)	26 (5.3)	4 (0.8)	NR	40 (8.2)	NR	NR	NR	NR	NR	NR	NR	NR	NR	8 (1.6)	NR	NR	NR	NR	NR	NR	NR	NR	NR	NR	NR	NR	NR	NR	NR	NR
	Negative COVID-19	14 (1.5)	48 (5.0)	15 (1.6)	NR	95 (9.9)	NR	NR	NR	NR	NR	NR	NR	NR	NR	27 (2.8)	NR	NR	NR	NR	NR	NR	NR	NR	NR	NR	NR	NR	NR	NR	NR	NR
Cruz-Lemini et al., 2021 [20]	Positive COVID-19	0	2 (1.1)	1 (0.6)	NR	2 (1.1)	5 (2.9)	NR	5 (2.9)	NR	NR	NR	9 (5.2)	NR	65 (38)	NR	NR	NR	NR	NR	94 (54.0)	12 (6.9)	86 (50.6)	84 (49.4)	NR	NR	NR	NR	39.0 (31–42)	NR	21 (12.1)	NR
	Negative COVID-19	0	2 (0.5)	3 (0.7)	NR	12 (2.8)	22 (5.1)	NR	7 (1.6)	NR	NR	NR	14 (3.3)	NR	177 (41.6)	NR	NR	NR	NR	NR	264 (61.4)	19 (4.4)	205 (48.2)	220 (51.8)	NR	NR	NR	NR	39.1 (28–42)	NR	55 (12.8)	NR
Li et al., 2020 [21]	Positive COVID-19	NR	NR	NR	NR	NR	NR	NR	NR	NR	NR	NR	NR	NR	NR	NR	NR	NR	NR	NR	NR	14 (87.5)	NR	NR	NR	NR	3066.7 (560.2)	NR	38 (0.2)	NR	NR	NR
	Negative COVID-19	NR	NR	NR	NR	NR	NR	NR	NR	NR	NR	NR	NR	NR	NR	NR	NR	NR	NR	NR	NR	16 (88.9)	NR	NR	NR	NR	3198.7 (522.6)	NR	38 (2.9)	NR	NR	NR
	Control 2020	NR	NR	NR	NR	NR	NR	NR	NR	NR	NR	NR	NR	NR	NR	NR	NR	NR	NR	NR	NR	57 (47.1)	NR	NR	NR	NR	3317.1 (455.3)	NR	39 (0.7)	NR	NR	NR
	Control 2019	NR	NR	NR	NR	NR	NR	NR	NR	NR	NR	NR	NR	NR	NR	NR	NR	NR	NR	NR	NR	44 (36.4)	NR	NR	NR	NR	3307.9 (419.3)	NR	38 (6.9)	NR	NR	NR
Melguizo et al., 2021 [22]	Positive COVID-19	26 (1.9)	19 (1.5)	15 (1.1)	5 (0.4)	52 (4.0)	NR	97 (7.4)	50 (3.7)	NR	NR	NR	48 (3.7)	NR	516 (38.7)	25 (1.9)	NR	NR	NR	NR	NR	NR	NR	NR	NR	NR	NR	NR	NR	NR	131 (10.2)	NR
	Negative COVID-19	28 (1.7)	17 (1.1)	11 (0.7)	5 (0.3)	52 (3.4)	NR	136 (8.6)	55 (3.4)	NR	NR	NR	44 (2.8)	NR	644 (40.4)	34 (2.1)	NR	NR	NR	NR	NR	NR	NR	NR	NR	NR	NR	NR	NR	NR	193 (12.8)	NR
Norman et al., 2021 [23]	Positive COVID-19	NR	NR	NR	NR	NR	NR	163 (7.0)	NR	NR	NR	NR	NR	NR	1001 (43.1)	76 (3.3)	NR	1321 (56.9)	NR	NR	1828 (78.7)	495 (21.3)	1157 (49.8)	1166 (50.2)	NR	NR	3436 (628)	NR	274.7 (15.1)	NR	55 (2.4)	2201 (97.6)
	Negative COVID-19	NR	NR	NR	NR	NR	NR	4331 (5.0)	NR	NR	NR	NR	NR	NR	37058 (43.2)	2304 (2.7)	NR	48740 (56.8)	NR	NR	70386 (82.0)	15450 (18.0)	43839 (51.1)	41997 (48.9)	NR	NR	3502 (559)	NR	277.0 (12.6)	NR	3048 (3.7)	79820 (96.3)
	Negative Covid (Matched cohort)	NR	NR	NR	NR	NR	NR	592 (6.4)	NR	NR	NR	NR	NR	NR	3995 (43.1)	287 (3.1)	NR	5278 (56.9)	NR	NR	7525 (81.1)	1750 (18.9)	4776 (51.5)	4499 (48.5)	NR	NR	3495 (558)	NR	277.1 (12.6)	NR	195 (2.1)	8887 (97.9)
Steffen et al., 2021 [24]	Population	172 (17.2)#	89 (8.9)	NR	NR	133 (13.3)	NR	NR	98 (9.8)	NR	NR	NR	NR	NR	NR	NR	NR	NR	NR	NR	NR	NR	NR	NR	NR	NR	NR	NR	39 (37–39)	NR	NR	NR
	Positive COVID-19	8 (13.7)#	4 (6.6)	NR	NR	4 (6.6)	NR	NR	4 (6.6)	NR	NR	NR	NR	NR	NR	NR	NR	NR	NR	NR	NR	NR	NR	NR	NR	NR	NR	NR	39 (37–39)	NR	NR	NR
	Negative COVID-19	147 (15.6)#	85 (9.1)	NR	NR	129 (13.7)	NR	NR	94 (10)	NR	NR	NR	NR	NR	NR	NR	NR	NR	NR	NR	NR	NR	NR	NR	NR	NR	NR	NR	39 (37–39)	NR	NR	NR
Tadas et al., 2021 [25]	Positive COVID-19	NR	NR	NR	NR	NR	NR	NR	NR	NR	NR	NR	NR	NR	NR	NR	69 (38.12)	112 (61.88)	NR	NR	NR	NR	NR	NR	NR	NR	NR	NR	38.19 (2.28)	NR	NR	NR
	Negative COVID-19	NR	NR	NR	NR	NR	NR	NR	NR	NR	NR	NR	NR	NR	NR	NR	79 (43.64)	102 (56.35)	NR	NR	NR	NR	NR	NR	NR	NR	NR	NR	38.14 (2.44)	NR	NR	NR
Taghavi et al., 2021 [26]	Positive COVID-19	5 (9.09)	3 (5.45)	NR	NR	3 (5.45)	NR	4 (7.27)	2 (3.63)	NR	NR	NR	NR	NR	15 (27.27)	NR	NR	NR	NR	NR	NR	NR	NR	NR	NR	NR	NR	NR	NR	NR	1 (1.81)	NR
	Negative COVID-19	2 (3.63)	2 (3.63)	NR	NR	0	NR	2 (3.63)	1 (1.81)	NR	NR	NR	NR	NR	12 (21.81)	NR	NR	NR	NR	NR	NR	NR	NR	NR	NR	NR	NR	NR	NR	NR	2 (3.63)	NR
Timircan et al., 2021 [27]	Positive COVID-19	NR	NR	NR	NR	NR	NR	NR	NR	NR	NR	NR	NR	NR	NR	NR	NR	NR	NR	NR	71 (70)	30 (30)	NR	NR	NR	NR	NR	NR	NR	NR	NR	NR
	Negative COVID-19	NR	NR	NR	NR	NR	NR	NR	NR	NR	NR	NR	NR	NR	NR	NR	NR	NR	NR	NR	779 (83)	159 (17)	NR	NR	NR	NR	NR	NR	NR	NR	NR	NR
Villar et al., 2021 [28]	Positive COVID-19	33 (4.7)	NR	9 (1.3)	5 (0.7)	NR	NR	NR	NR	NR	NR	NR	NR	NR	NR	NR	NR	NR	NR	NR	NR	NR	NR	NR	NR	NR	NR	NR	NR	NR	NR	NR
	Negative COVID-19	20 (1.4)	NR	27 (1.9)	16 (1.1)	NR	NR	NR	NR	NR	NR	NR	NR	NR	NR	NR	NR	NR	NR	NR	NR	NR	NR	NR	NR	NR	NR	NR	NR	NR	NR	NR
